# Loss of function of miR-342-3p results in MCT1 over-expression and contributes to oncogenic metabolic reprogramming in triple negative breast cancer

**DOI:** 10.1038/s41598-018-29708-9

**Published:** 2018-08-16

**Authors:** Sandra L. Romero-Cordoba, Sergio Rodriguez-Cuevas, Veronica Bautista-Pina, Antonio Maffuz-Aziz, Elvira D’Ippolito, Giulia Cosentino, Sara Baroni, Marilena V. Iorio, Alfredo Hidalgo-Miranda

**Affiliations:** 1 Cancer Genomics Laboratory, National Institute of Genomic Medicine, Mexico City, Mexico; 2 Instituto de Enfermedades de la Mama FUCAM, Mexico City, Mexico; 3 Start Up Unit, Department of Experimental Oncology and Molecular Medicine, Fondazione IRCCS Istituto Nazionale dei Tumori, Milan, Italy

## Abstract

Triple-negative breast cancer (TNBC) is a heterogeneous and aggressive neoplasia lacking the expression of hormonal receptors and human epidermal growth factor receptor-2. Accumulating evidence has highlighted the importance of miRNAs dysregulation in the establishment of cancer programs, but the functional role of many miRNAs remains unclear. The description of miRNAs roles might provide novel strategies for treatment. In the present work, an integrated analysis of miRNA transcriptional landscape was performed (N = 132), identifying the significant down-modulation of miR-342-3p in TNBC, probably because of the aberrant activity of estrogen receptor, which serves as a transcription factor of the miRNA, as demonstrated by a siRNA-knockdown approach. The enhanced expression of miR-342-3p significantly decreased cell proliferation, viability and migration rates of diverse TN cells *in vitro*. Bioinformatic and functional analyses revealed that miR-342-3p directly targets the monocarboxylate transporter 1 (MCT1), which promotes lactate and glucose fluxes alteration, thus disrupting the metabolic homeostasis of tumor cells. Optical metabolic imaging assay defined a higher optical redox ratio in glycolytic cells overexpressing miR-342-3p. Furthermore, we found that hypoxic conditions and glucose starvation attenuate miR-342-3p expression, suggesting a crosstalk program between these metabolic factors. Consistently, miR-342-3p down-modulation is associated with an increased MCT1 expression level and glycolytic score in human triple negative tumors. Overall, we described for the first time the regulatory activity of miR-342-3p on relevant metabolic carcinogenic pathways in TN breast cancers.

## Introduction

Breast cancer remains one of the most common neoplasia exhibiting a substantial diversity of histological and molecular features. Breast cancer classification is predominantly based on immunohistochemical assay evaluating the presence of hormone, estrogen (ER) and progesterone (PR) receptors, and the human epidermal growth factor receptor (HER2). Triple-negative breast cancer (TNBC) is an aggressive tumor subtype lacking ER, PR and HER2 expression, having limited target therapy options that result in aggressive clinical outcomes^1,2^.

Over the past years, several studies have revealed that miRNA expression is dysregulated in human malignances, contributing to cancer hallmarks through the post-transcriptional regulation of target genes, promoting their degradation or translational repression^3^^.^ MiRNAs serve as oncogenes or tumor suppressors regulating initiation and progression programs^4,5^. Moreover, well-known miRNAs impact metabolic pathways to sustain tumor biosynthetic needs^6–8^.

In tumors, the rate of glucose uptake dramatically increases and bypasses ATP production through oxidative phosphorylation, switching to a non-oxidative pathway by aerobic glycolysis and lactate production^9^. ATP molecule production per glucose molecule is limited in aerobic glycolysis, consequently cancer cells increase the uptake of glucose molecules from extracellular space to meet energy requirements, and secrete more lactic acids into the microenvironment to maintain cellular homeostasis. Lactate fluxes are mainly maintained by monocarboxylate transporter (MCT), particularly MCT1^10^. In fact, new pharmacological approaches have been developed to alter the aerobic glycolysis by MCT inhibitors, thus altering lactate fluxes^11^.

A more complete analysis of miRNA expression in different tumors and metabolic scenarios will improve our understanding of how different pathways are affected in cancer, particularly to address how miRNA dysregulation contributes to determinate the biological and metabolic needs of the tumors. In this study, we analyzed miRNA expression of 132 TN tumors by in-house profiling, as well as public available data to deeply characterize their miRNA expression landscape. Our data identified the reduced expression of miR-342-3p in triple negative tumors and its association with poor prognosis. We also identified a significant correlation between ER loss of function and the down-modulation of miR-342-3p in TN tumors. Furthermore, our molecular and transcriptional characterization of the biological function of miR-342-3p led us to conclude that re-expression of miR-342-3p significantly suppresses proliferation, viability and migration in *in vitro* models. MCT1 was identified as a putative target of this miRNA, and the down-modulation of this molecule resulted in altered metabolic program of cancer cells, especially in altered lactate-glucose fluxes. We hypothesized that the reduced expression of miR-342-p in TN tumor could be involved in the pathogenesis of this breast cancer subtype through the regulation of important cancer programs, such as proliferation and lactate metabolism.

## Results

### miRNA landscape across TNBC expression profiles

**Figure 1:**
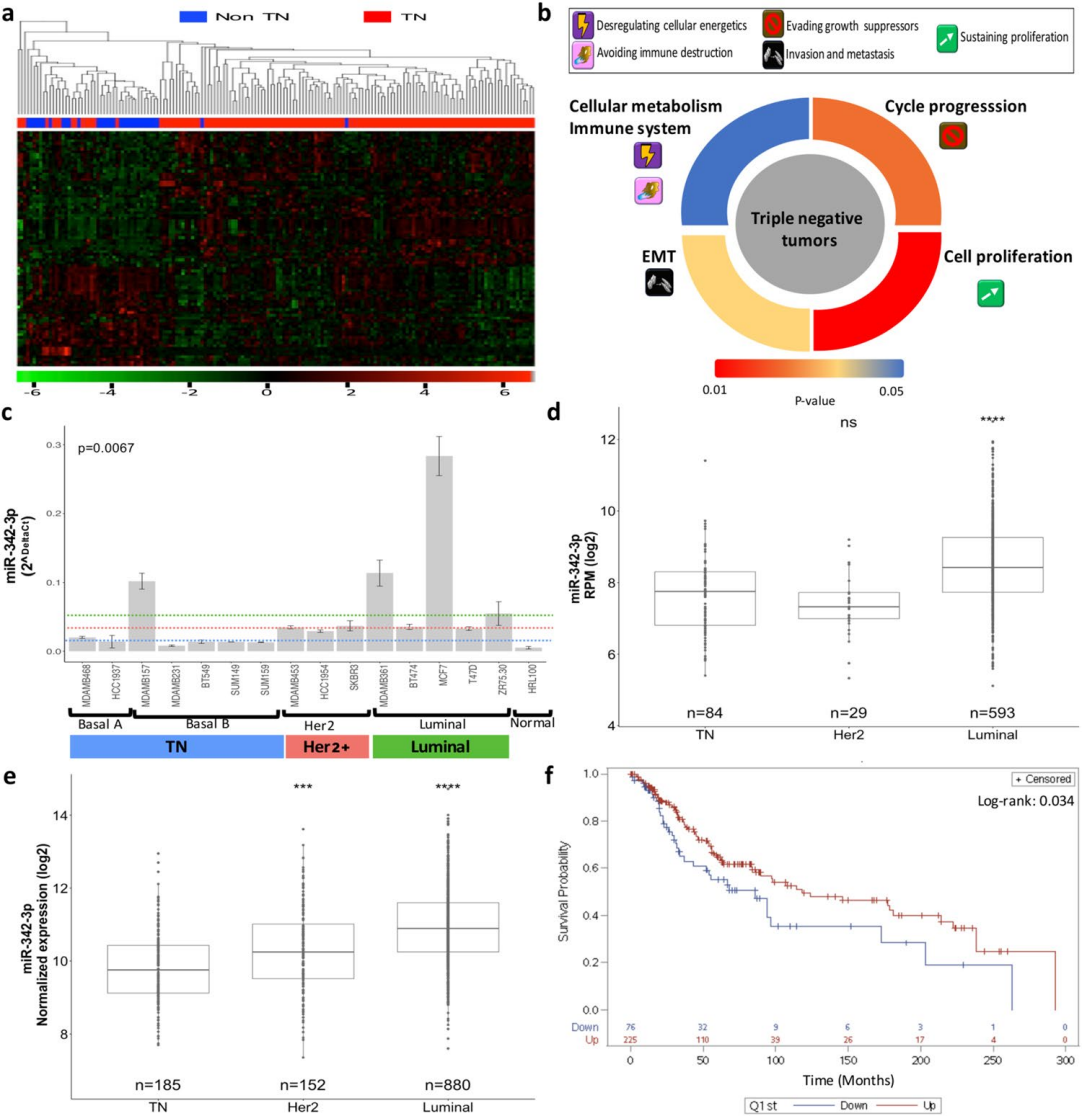
miR-342-3p is down-modulated in TN tumors and associated to oncogenic features and poor clinical outcomes. (**a**) Heatmap and hierarchical clustering of miRNAs differentially expressed between TN tumors vs other phenotypes. (**b**) Pathway enrichment analysis of miRNAs-mRNAs altered in triple negative tumors. (**c**) RT-qPCR expression of miR-342-3p in breast cancer cell lines. The panel of cell lines showed a heterogeneous miR-342-3p expression with a significant down-modulation in basal A and B cell lines. Kruskal-Wallis test showed the statistical significance between the multiple phenotype comparisons. Expression level of miR-342-3p in (**d**) TCGA and (**e**) METABRIC databases across IHC subtypes, TN tumors: ER−, PR− Her2−, Her2 tumors: ER−, PR− and Her2+ and luminal tumors: ER/PR+ , Her2− or ER/PR+, Her2+ (**f**) Survival analysis according to miR-342-3p expression. Lower miR-342-3p expression levels is associated with a decreased overall survival in patients with triple negative breast cancer. Kaplan-Meier survival curve shows patients with lower miR-342-3p expression (≤1st quantile) in blue line and higher miR-342-3p expression (>to 1st quantile) in red line.

We first evaluated the miRNA landscape of TN tumors compared with other tumor phenotypes (TNBC N = 132, ER + and/or PR + and/or Her2 + , N = 32). Our genomic approach revealed 83 differentially expressed miRNAs (Fig. 1a, Table S1). Integrative analysis of the altered mRNA-miRNA expression patterns of the same profiled tumor identified a significant enrichment of processes related to cell cycle progression, cell proliferation, epithelial mesenchymal transition and cellular metabolism in triple negative tumors (Fig. 1b, Table S2). These results explain the aggressive phenotype of TN cancers, but they also indicate how the altered expression of miRNAs contributes to the oncogenic pathways that promote tumor progression and stabilization.

Among the most significant down-modulated miRNAs, we identified miR-342-3p (lgFC: −1.51, adjusted p-value > 0.001), which has been already reported by different miRNA profiles as down-modulated in TNBC^12–14^. However, its biological function is not completely understood. MiR-342-3p expression was also expressed at lower levels in different TN cell line models (Kruskal-Wallis p-value: 0.0067, Fig. 1c), indicating that its down-regulation is important in the TN phenotype. To independently validate the down-modulation of miR-342-3p in TN tumors, we analyzed public data bases (TCGA - https://xenabrowser.net/ - and METABRIC^15^), which include a total of 280 TN tumors, confirming its reduced expression in this tumor subgroup compared with other phenotypes (Fig. 1d,e). In addition, the reduced expression of miR-342-3p in TN tumors is significantly associated with a poor clinical prognosis in triple negative tumors (Fig. 1f).

### miR-342-3p expression is modulated by estrogen receptor

**Figure 2:**
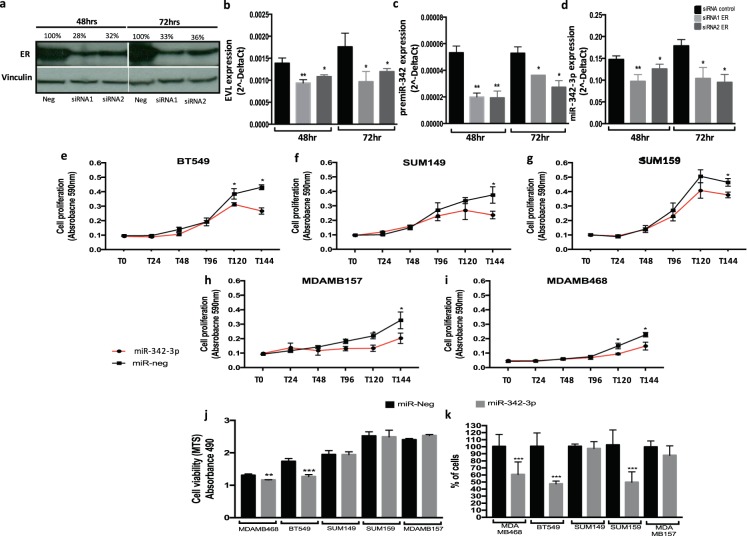
miR-342-3p is regulated by ER and its exogenous expression down-modulates oncogenic pathways in TN cells. (**a**) MCF7 cell line transfected with siRNA-mediated sequences against ER. Evaluation of the down-modulation of ER by an immunoblotting analysis after 48 and 72 hr of siRNA transfection. Densitometric analysis indicated as percentage above the blot (the blot is representative of 3 independent experiments). Real-time qRT-PCR evaluation in MCF7 cells transfected with ER siRNAs at 48 and 72 hr post-transfection of (**b**) EVL, (**c**) pre-miR-342 and (**d**) miR-342-3p. Cell growth rate evaluation by SRB assay after 144 h in (**e**) BT549, (**f**) SUM149, (**g**) SUM159, (**h**) MDAMB157 and (**i**) MDAMB468. (**j**) Cell viability evaluated by MTS assay at 48 h post-transfection, (**k**) cell migration evaluated by a transwell assay on TNBC cells transfected with miR-342-3p precursor or control for 48 h. Results of three independent experiments with three technical replicates are represented as the geometric mean +/− standard deviation error.

A potential modulator of miR-342-3p expression is the estrogen receptor (ER)^12,13,16^, which serves as a transcription factor of several genes. We hypothesized that the miRNA down-modulation in this tumor type is a consequence of the absence of ER expression. MiR-342-3p is an intronic miRNA of the EVL gene, which is also down-modulated in TN tumors and its expression is regulated by ER activity^17^. An *in-silico* analysis of the genomic architecture of miR-342-3p sequence did not identify any alternative promoter based on histone marks or polimerase II enrichments, so we concluded that the expression of miR-342-3p depends on the regulatory sequences and expression of the host gene EVL. Bioinformatics analyses further support the association of ER with miR-342-3p/EVL through the detection of two proximal binding sites for ER in the EVL promoter, validated by public ER chromatin immunoprecipitation-sequencing data (ChIP-seq. 1 kb) in ER positive cell line models (http://chip-atlas.org/) (Sup Fig. 1). Furthermore, we corroborated the positive correlation between EVL and miR-342-3p expression (Sup Fig. 2a,b), as well as the correlated expression status of ER, EVL and miR-342-3p in breast tumors reported in public databases (Sup Fig. 2c–f). Finally, to functionally validate these observations, ER was silenced by two different siRNA-mediated sequences in the MCF7 cell line (ER+, PR+ and Her2−) (Fig. 2a). The expression of EVL (Fig. 2b), premiR-342 (Fig. 2c) and miR-342-3p (Fig. 2d) were significantly decreased by ER knock-down.

### miR-342-3p suppresses cell growth, viability and migration in triple negative cell line models

To determine the functional significance of miR-342-3p in TN cell models, we evaluated the impact of its exogenous expression on TN tumor cells. The exogenous over-expression of miR-342-3p significantly reduced growth rate in all the cell line models evaluated (BT549, SUM149, SUM159, MDAMB157 and MDAMB468) (Fig. 2e–i), whereas cell viability was decreased (Fig. 2j) in a significant proportion of cells analyzed (MDAMB468 and BT549). Impairment of cell migration by exogenous expression of miR-342-3p was also observed in the cell lines MDAMB468, BT549 and SUM159 (Fig. 2k), whereas invasion and apoptosis were not significantly affected by the miRNA (data not shown). These data suggest a possible tumor suppressor activity of miR-342-3p in triple negative cell line models.

### MCT1 is a direct target of miR-342-3p

**Figure 3:**
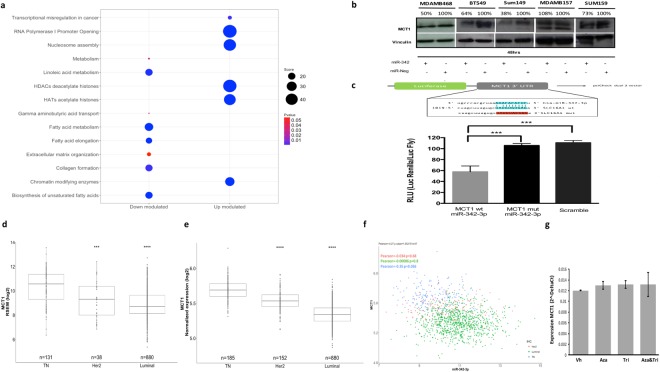
MiR-342-3p regulates the expression of its putative target MCT1. (**a**) Pathway enrichment analysis of the differentially expressed genes after miR-342-3p transfection (48 hr) in MDAMB468 cells, performed with Enrichr database using KEGG, Reactome and Gen Ontologies functional annotations. (**b**) MCT1 protein expression at 48 hr post-transfection of miR-342-3p in TN cell lines by an immunoblotting analysis. Densitometric analysis is indicated as percentage above the blot (the blot is representative of 3 independent experiments). Plot figure is a group of blots cropped from different gels, complete and original blots are shown in Sup Fig. 4 (**c**) MDAMB468 cells were co-transfected with: miR-342-3p, psi-Check dual-luciferase system containing the miRNA predicted binding site of the MCT1 3′UTR and one mutated form (Mut1). Renilla luciferase activity was measured 24 hr after transfection and normalized on Firefly luciferase activity. Expression level of MCT1 in TCGA (**d**) and Metabric (**e**) database subgrouped by IHC subtypes, TN tumors: ER−, PR− Her2−, Her2 tumors: ER−, PR+ and Her2+ and luminal tumors: RE+, RP+/− and HER2 −/+. (**f**) Correlation of MCT1 and miR-342-3p expression levels in breast cancer (Metabric data). (**g**) qRT-PCR evaluation of miR-342-3p expression level after 5-azacytidine and/or trichostatin treatment on MDADB468 cells. Results of three independent experiments with three technical replicates are represented as the geometric mean +/− standard deviation error.

To understand the mechanism by which the down-modulation of miR-342-3p impacts cancer programs, we performed an exploratory analysis on the transcriptomic landscape of MDAMB468 after the miRNA over-expression. This analysis showed a total of 103 altered mRNAs, and among them 12 genes were identified as putative targets of the miRNA, defined by an *in-silico* analysis, of which 6 had been predicted from at least 3 different algorithms and had been confirmed as altered transcripts in triple negative tumors by TCGA profiles data (Table S2). Remarkably, the ontological enrichment of the altered mRNAs resulted by the miR-342-3p gain of function identified important pathways related to key functions in tumors cells, such as the up-modulation of nucleosome assembly and chromatin-histones modifying enzymes, as well as the down-modulation of collagen formation, extracellular matrix organization, gamma acid transports, fatty acid metabolism and cellular metabolism (Fig. 3a, Table S3). For instance, MCT1 (SLC16A1), a predicted direct target of miR-342-3p (Table S3), acts as a monocarboxylate transporter which serves as the main lactate transporter in breast cancer cells and its over-expression has been associated with poor clinical outcomes^18–20^. Altered metabolism has been recognized as a hallmark of cancer since the metabolic reprogramming improves cellular fitness^21,22^, thus a careful identification of deregulated metabolism via the regulatory network of miR-342-3p and MCT1 emerge as an interesting mechanism to be deeply characterized.

To confirm the direct inhibition of MCT1 by miR-342-3p, we evaluated its protein expression level after the exogenous expression of the miRNA in TN cell lines. The restoration of miR-342-3p down-modulated MCT1 expression in the TN cell line models: MDAMB468, BT549, SUM149 and SUM159 (Fig. 3b). Finally, a reporter luciferase assay on MDAMB468 cells exogenously over-expressing miR-342-3p and co-transfected with a reporter plasmid fused with the 3′UTR binding sequence of MCT1 showed a significant suppression of the luciferase activity (Fig. 3c). The inhibition of the luciferase activity was abolished by mutations in the seed region of the miRNA binding site (Fig. 3c). In conclusion, these data demonstrate that MCT1 is directly targeted by miR-342-3p in diverse TN cell line models.

### The expression of MCT1 is negatively correlated with the expression of miR-342-3p in TN tumors

To identify a potential correlation between miR-342-3p and MCT1 in human TN tumors, we analyzed gene expression profiling data from TCGA and Metabric. The differential expression of MCT1 among breast cancer subtypes was validated, finding an up-modulation in TN tumors (Fig. 3d,e). Notably, we found a significant anti-correlation between miR-342-3p and MCT1 expression in the TN phenotype (Fig. 3f Pearson = −0.35, p.value = 0.036).

Furthermore, alternative mechanisms for the up-modulation of MCT1 in TN tumors were explored in the TCGA database^23^. We did not find any significant mutation, copy number variation or de-methylation event which might explain the up-regulation of MCT1, so we concluded that miR-342-3p constitutes one of the principal regulatory mechanisms of MCT1 expression in TN cancer. To further confirm this finding in our experimental model (cell line MDAMB468), we performed a 5-azacytidine and tricostatin treatment without any significant change in MCT1 expression after treatment, so we can exclude the methylation or histone acetylation as possible regulatory mechanisms of the target gene, confirming our previous results (Fig. 3g).

### miR-342-3p decreases the consumption of exogenous lactate

**Figure 4:**
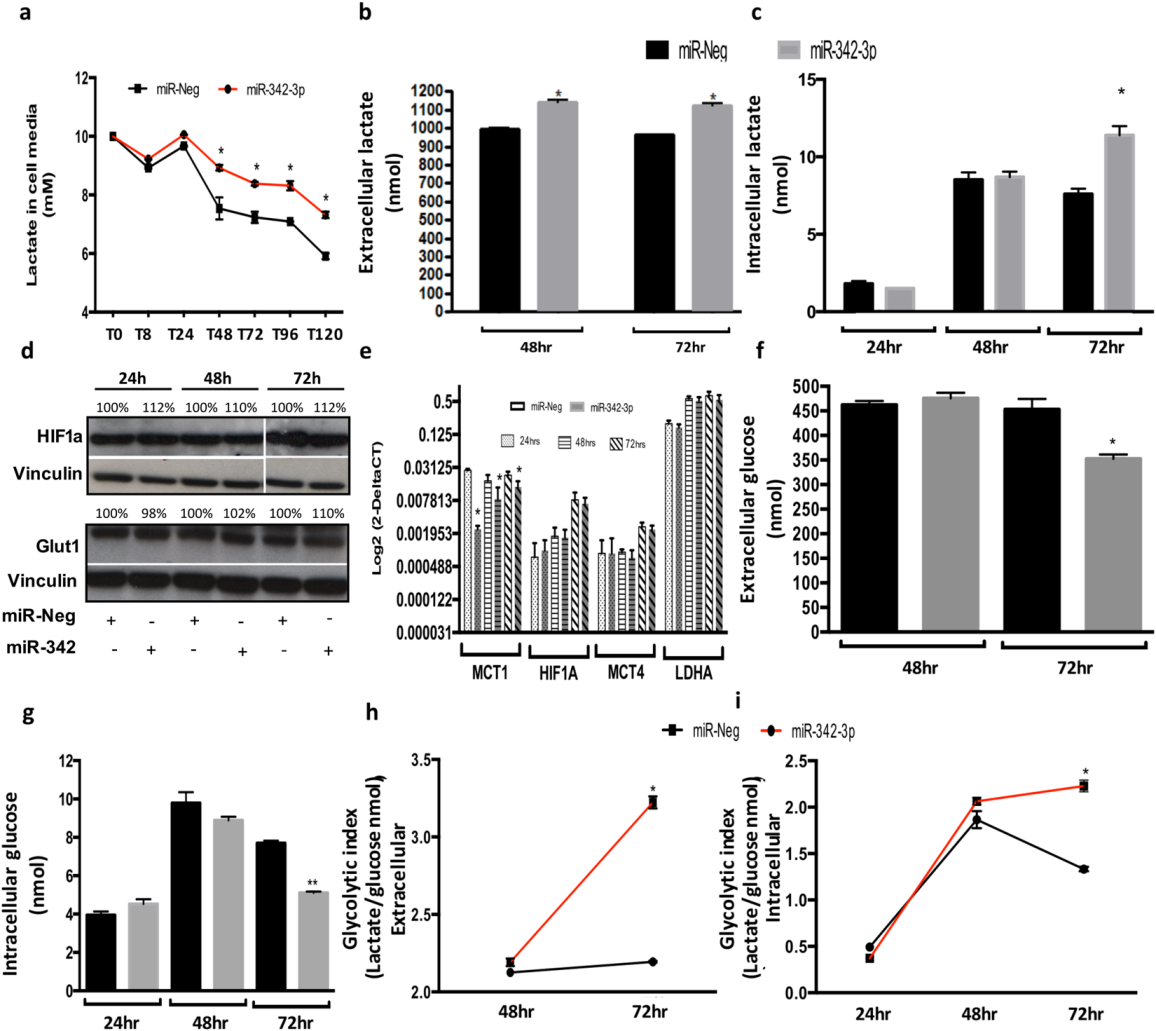
Exogenous expression of miR-342-3p disrupts extra and intracellular lactate concentrations, and glucose levels. (**a**) Evaluation of exogenous lactate (10 mM) consumption of MDAMB468 cells transfected with miR-342-3p precursor quantified by an enzymatic colorimetric assay (cells were cultured in low glucose medium). Enzymatic colorimetric assay of endogenous lactate concentration of MDAMB468 cells transfected with miR-342-3p precursor or control in (**b**) extracellular (medium) or (**c**) intracellular compartments. (**d**) Evaluation by immunoblotting analysis of HIF1A and GLUT1 protein expression at 24, 48 and 72 hr post-transfection of miR-342-3p precursor in MDAMB468 cells. Densitometric analysis is indicated as percentage above the blot. (**e**) qRT-PCR assay of MCT1, HIF1A, MCT4 and LDHA expression on MDAMB468 cells transfected with miR-342-3p precursor. Enzymatic colorimetric assay of endogenous glucose concentration of MDAMB468 cells transfected with miR-342-3p precursor or control in (**f**) extracellular or (**g**) intracellular compartments. Glycolytic index of cells exogenously transfected with miR-342-3p compared to control in (**h**) extracellular and (**i**) intracellular compartments. The plots are representative of three independent experiments with three technical replicates, each summarized as the geometric mean +/− standard deviation error.

Since the principal role of MCT1 is the lactate transportation from the extracellular space into the cell, we evaluated exogenous lactate consumption in low glucose medium after miR-342-3p transfection. The used concentration of the extracellular lactate (10 mM sodium lactate) mimics the quantity of lactate detected in tumors^24^. MDAMB468 TN cell line was selected as a functional model since it presents an active glycolytic phenotype and a membrane expression of MCT1 and MCT4^25^, important monocarboxylate transporters. In particular, MCT1 is the predominant regulator of lactate fluxes, as indicated by its affinity constant (Km = 3–6 mM), while MCT4 presents less affinity for lactate (km = 25–30 mM)^26^. Thus, in presence of lactate but not glucose, MDAMB468 exogenously over-expressing miR-342-3p diminished their lactate consumption (Fig. 4a), without any significant change in the pH of the medium. Our data suggest that the down-modulation of MCT1 by miR-342-3p abolishes the incorporation of lactate into the cells, promoting its accumulation in the cell culture medium.

### Intra and extracellular lactate and glucose concentrations are altered by MCT1 inhibition via miR-342-3p

The endogenous lactate and glucose levels, measured by enzymatic assays, were evaluated in the cell culture media and in the tumor cells. The extracellular lactate concentration (cellular media) increased upon 48 and 72 hr post-transfection of miR-342-3p (Fig. 4b), suggesting that the accumulation of lactate outside the cells might be a consequence of the decreased expression of the lactate transporter MCT1 induced by miR-342-3p. Surprisingly, we also noticed an increase of intracellular lactate concentration in comparison to control cells after 72 h of miR-342-3p transfection (Fig. 4c). This effect can be attributed to an increase in the lactate production, but not to a higher internalization of the metabolite, since we observed an extracellular lactate accumulation as consequence of MCT1 inhibition.

To rule out other potential mechanisms that may impact the production and fluxes of lactate, we analyzed HIF1A (Fig. 4d,e), which under hypoxic conditions or high lactate concentrations promotes the expression of MCT4^27^, another lactate transporter with lower affinity than MCT1^28^, as well as LDHA, which allows the conversion of pyruvate to lactate^29^. However, none of these genes presented a significant over-expression in miR-342-transfected cells (Fig. 4e).

To have a better image of the metabolic activity of the cells over-expressing miR-342-3p, we also evaluated the levels of intra and extracellular glucose, to define if there are changes in other energetic molecules acting as substrates for ATP production. Glucose levels were decreased in the cell culture media and inside the cells 72 hr post-transfection (Fig. 4f,g). To exclude alternative mechanisms which might result in glucose level alterations, we measured the expression of one of the most active glucose transporters, Glut1, which did not show any significant change in its expression among the experimental conditions (Fig. 4d). We can conclude that exogenous expression of miR-342-3p, enhance glucose cell consumption over lactate, since cells are not able to introduce it. Thus, glucose becomes the mayor energetic source, which is probably converted to lactate via glycolysis.

Having assessed lactate and glucose levels, we noticed that the extracellular glycolytic index is higher in cells over-expressing miR-342-3p at 72 hr post-transfection (Fig. 4h), and the same behavior was detected at the intracellular compartment (Fig. 4i). Thus, the rate of glucose consumption resulted in a higher intracellular lactate production.

These observations suggest that the expression of miR-342-3p promotes an accumulation of extracellular lactate through MCT1 inhibition, which might result in an increased consumption of glucose, as shown by the extra and intra-cellular reduced level of this metabolite. The glucose is then oxidized in the cell via glycolysis, generating lactate as a sub-product, which is increased in the cells transfected with the miRNA.

### miR-342-3p rescue disrupts metabolic homeostasis impairing glycolysis and oxidative phosphorylation in TNBC cells

**Figure 5:**
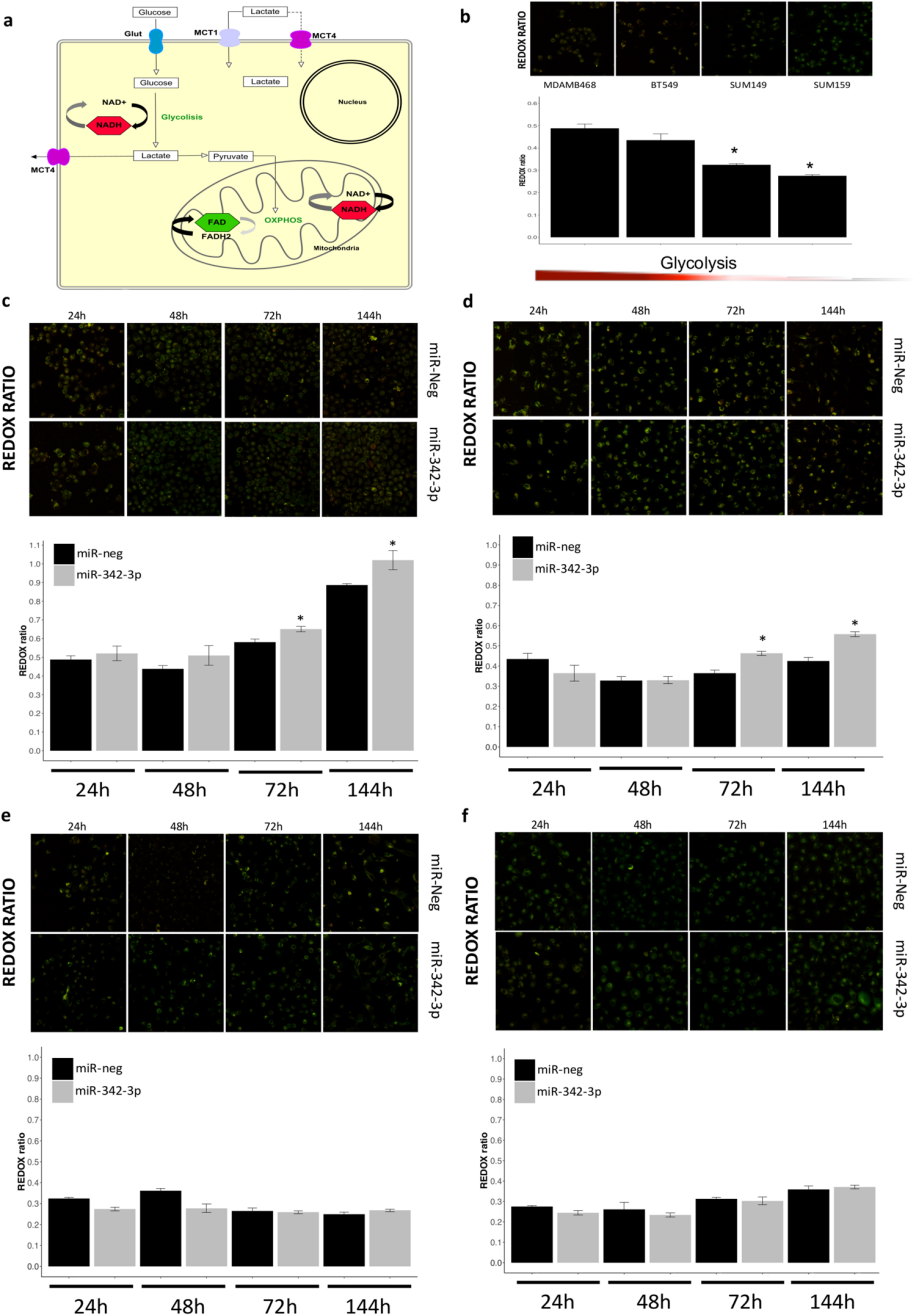
Over-expression of miR-342-3p disrupts redox ratio homeostasis in living TN cells (**a**) Graphical representation of glycolysis and OXPHOS process on cells as well as the auto-fluorescent NADH and FAD molecules. (**b**) Representative images of the optical redox ratio of living cells MDAMB468, BT549, SUM149, SUM159 exogenously expressing miR-342-3p or control at 24, 48, 72 and 144 hr post-transfection. Among the cell line panel evaluated, there was a decreasing gradient in glycolytic state. Redox ratio (NADH/FAD) of (**c**) MDAMB468, (**d**) BT549, (**e**) SUM149 and (**f**) SUM159 cells transfected with miR-342-3p precursor or control. The stack series were background corrected and total integrative fluorescent intensity was measured by single cell. Results were summarized with a geometric mean and standard deviation error of the stacks per time and condition. The RGB image is a Z projection using a sum slide projection of the stack (only for image presentation). Statistical significance was computed with a Wilcoxon test comparing the mean redox ratio value of miR-342-3p transfected cells vs control samples for each time.

An important adaptation of tumors is the metabolic symbiosis with other tumoral and stromal cells, which allows tumor adjustment to drastic energetic changes. We observed that the down-modulation of miR-342-3p is involved in the metabolic crosstalk of TN cells, promoting the establishment of a metabolic homeostasis between cells consuming glucose and lactate as energetic source. To extend our understanding of how miR-342-3p impacts metabolic profiling we evaluated the redox ratio of living cells exogenously expressing miR-342-3p by an optical metabolic imaging (OMI) assay, which analyzed the auto-fluorescent intensity of the metabolic enzymes: reduced nicotinamide adenine dinucleotide (NADH) and flavin adenine dinucleotide (FAD). In summary, NADH is produced during glycolysis as the result of NAD+ reduction, whereas the pyruvate resulting from glycolysis is incorporated into the mitochondria to react in the oxidative phosphorylation (OXPHOS). This process consumes NADH and generates FAD which acts as the principal electron donor^30^ (Fig. 5a). The OMI assay only detected auto-fluorescence emitted by NADH and FAD since their oxidized (NAD+) and reduced (FADH2) forms do not emit any fluorescence^31^ (Fig. 5a). The sum of fluorescence intensities was used to compute the optical redox ratio, which measures the glycolysis level mirrored by the oxidized or reduced status of NADH or FAD^32^ and was computed as the ratio of total NADH fluorescence intensity divided by total FAD fluorescence intensity^30,33,34^. The metabolic effect of miR-342-3p up-modulation was investigated in four TN cell lines that present miR-342-3p-mediated post-transcriptional regulation of MCT1 (Fig. 3b) and heterogeneous metabolic status (Fig. 5b): MDAMB468, BT549, SUM159 and SUM149. MiRNA rescue expression raises the redox ratio due to an increase in glycolysis in MDAMBA468 and BT549, two of the most glycolytic cell models evaluated (Fig. 5c,d). In detail, a net loss of FAD fluorescent levels relative to NADH levels is observed in cells over-expressing miR-342-3p at 72 and 144 hours. On the other hand, SUM149 and SUM159 cells, the less glycolytic models, do not present any significant metabolic change after miR-342-3p over-expression in any time point (Fig. 5e,f).

**Figure 6:**
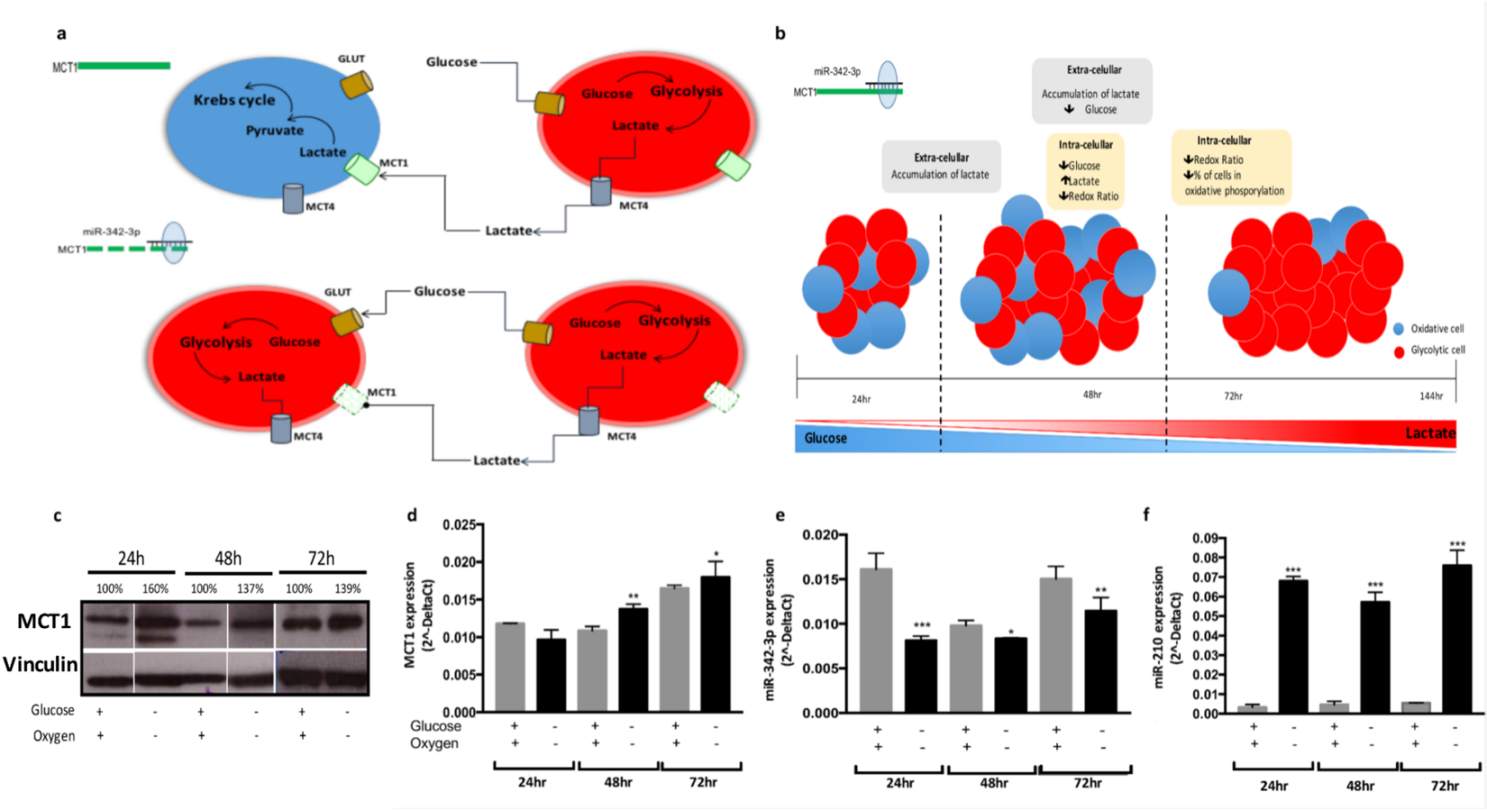
Feedback regulation circuit of metabolic phenotypes and miR-342-3p expression. (**a**) Graphical representation of the relation between miR-342-3p, MCT1 and lactate consumption in tumoral cells. TN tumors maintain a homeostatic relation between glycolytic and oxidative cells: glycolytic cells produce lactate as a bio-product, which is then exported to the medium through MCT transporters and used as an energetic source by oxidative cells, which incorporate it through MCT1 and convert it into pyruvate to then metabolize it via mitochondria. When miR-342-3p is over-expressed, it negatively regulates the expression of MCT1 disrupting the lactate fluxes, mainly the incorporation of lactate into the oxidative cells, promoting a metabolic change to a glycolytic state and establishing a competition for glucose. (**b**) Graphical representation of the hypothesized relation between the over-expression of miR-342-3p and metabolic states in the glycolytic MDAMB468 cells. The up-modulation of miR-342-3p expression in a glycolytic cell line model produces an accumulation of extracellular lactate by the inhibition of the lactate transporter MCT1, accompanied by an increment of intracellular lactate, while glucose presents a lower concentration in the transfected cells, together with a decrease of the redox ratio. A reduction in the number of oxidative cells and consequently an increment of glycolytic cells also occurred. (**c**) Immunoblots of MCT1 protein expression in MDAMB468 cells cultured under hypoxic conditions and glucose starvation or not after 24, 48 and 72 hr. Densitometric analysis is indicated as percentage in the blot. Plot figure is a group of blots cropped from different parts of the same gel, complete and original blots are shown in Sup Fig 4. qRT-PCR evaluation of (**d**) MCT1, (**e**) miR-342-3p and (**f**) miR-210 expression in MDAMB468 cells cultured in hypoxic conditions and under glucose starvation or not during 24, 48 and 72 hr. Results are representative of three independent experiments with three technical replicates, each summarized as the geometric mean +/− standard deviation error.

To summarize, miR-342-3p gain-of-function in cells presenting a more glycolytic phenotype disrupts the energetic fluxes between glycolytic and oxidative cells, promoting a switch from lactate oxidative phosphorylation to glycolysis, which eventually leads to a cellular competition for glucose intake with the final impairment of the metabolic homeostasis in TN cells (Fig. 6a,b).

### Low glucose and oxygen concentration decreases miR-342-3p and MCT1 expression

Limited glucose and oxygen bioavailability is a key feature of the tumor microenvironment^24^. Triple negative tumors have been described as glycolytic and hypoxic tumors, which are biological characteristics related with an aggressive malignant phenotype^35,36^. Since we demonstrated that miR-342-3p is a modulator of MCT1, it is possible that the metabolic conditions can also regulate the expression of miR-342-3p. Indeed, glucose deprivation upregulates MCT1 protein expression endowing an increased migration capability^37^. Therefore, we tested how glucose and oxygen levels modulate miR-342-3p and consequently MCT1 expression. Glucose-starved (1 mg/L) MDAMB468 cells growing in hypoxic condition (1%O_2_) presented a down-regulation of miR-342-3p, which triggers MCT1 expression at mRNA and protein level, whereas rescuing condition of normoxic (20–21% O_2_) and high-glucose (4 mg/L) concentration restores miR-342-3p expression and its post-transcriptional regulation over MCT1 (Fig. 6c–e). Likewise, as a control we evaluated the expression of the hypoxia-induced miR-210^38^, with a significant correlation between its up-regulation and hypoxic condition (Fig. 6f).

### Diverse metabolic and cellular enrichment in TN tumors expressing different levels of miR-342-3p

**Figure 7:**
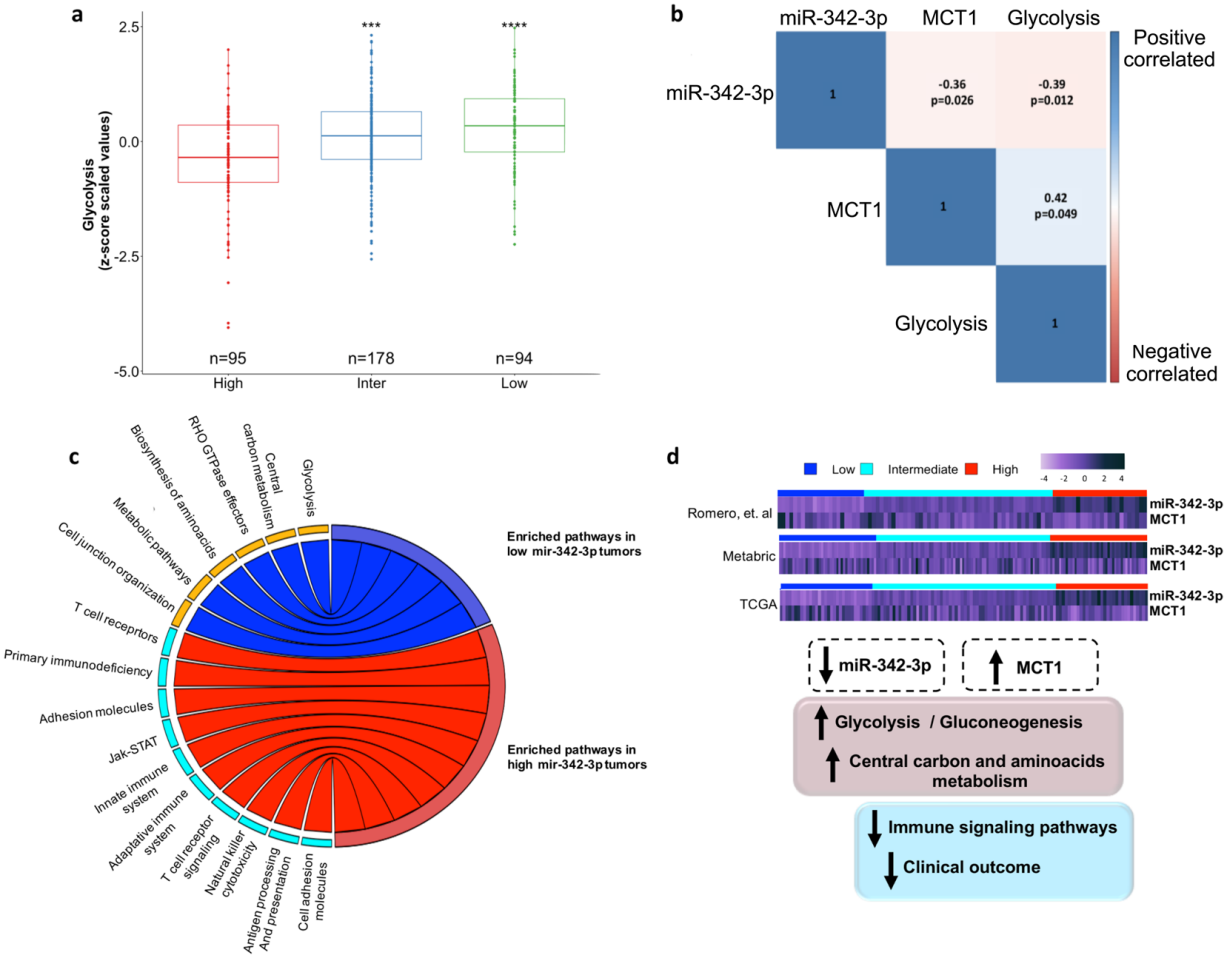
TN tumors expressing low levels of miR-342-3p are enriched in glycolysis pathway. (**a**) Z-score values of single sample GSEA analysis (ssGSEA) based on Glycolysis terms of TN tumors from our cohort, METABRIC and TCGA database stratified by miR-342-3p expression: low (≤1st quantile), intermediate (>1st quantile but <3^rd^ quantile) and high (≥3^rd^ quantile). (**b**) Correlation between miR-342-3p, MCT1 and glycolysis ssGSEA score (**c**) Chord plot illustrating the significant enriched pathways terms that are overrepresented in tumors with low miR-342-3p level (blue) and tumors with high miR-342-3p level (red) (tumors from our cohort, TCGA and METABRIC databases) (**d**) General conclusion of the descriptive genomics of TN tumors in accordance to miR-342-3p expression. Heatmap of the expression of miR-342-3p and its target gene MCT1 in profiled tumors (our cohort, METABRIC and TCGA) divided by miR-342- 3p expression level. The relative abundances of miR-342-3p and MCT1 are inversely based on miR-342-3p expression level.

To gain further insight into the metabolic state involving miR-342-3p expression in triple negative tumors, we applied a ssGSEA analysis to measure the overrepresentation of glycolytic metabolism (KEGG HSA00010) in each of the following cohorts: our tumor cohort (Romero cohort), METABRIC and TCGA data. To achieve a more confident view of the glycolytic metabolism of triple negative human cancers, we divided each cohort in three subgroups according to miR-342-3p expression levels: 1) low expression (<1^st^ quantile) 2) Intermediate (>1^st^ quantile <3^rd^ quantile) and 3) high (>3^rd^ quantile). The main divergence in the gene enrichment scores was associated with tumors presenting low or intermediate expression of miR-342-3p, characterized by a higher enrichment of glycolysis (Fig. 7a), as well as a significant up-modulation of anion transmembrane transport, in which MCT-mediated lactate transports are annotated (Sup Fig. 3).

We further examined the correlation of the expression levels of miR-342-3p, MCT1 and glycolysis phenotype. Levels of miR-342-3p were negatively correlated with MCT1 mRNA expression and glycolytic ssGSEA scores (Pearson: −0.36, p = 0.026, Pearson: −0.39, p = 0.012, respectively); in agreement with this observation MCT1 expression is positively correlated with glycolytic scores (Pearson: 0.042, p = 0.049) (Fig. 7b).

To identify signaling pathways potentially involved in miR-342-3p activity and TN tumorigenesis, a functional enrichment analysis was independently performed on our cohort, TCGA and METABRIC data bases between tumors presenting low expression levels of miR-342-3p vs high levels. Notably, relevant metabolic pathways resulted enriched in tumors down-expressing miR-342-3p such as glycolysis, carbon metabolism, amino acid biosynthesis and cell junction organizations in at least two tumor cohorts (Table S4). Expression of these set of genes is consistent with a glycolytic phenotype, which sustains the energetic requirements of tumor cells. In contrast, immune signaling, of both innate and adaptative immune system, was significantly enriched in tumors with higher levels of miR-342-3p, as well as the up-modulation of cell adhesion pathways (Fig. 7c, Table S4). We further characterized the immune phenotype of TNBC according to miR-342-3p expression by computing the immunophenoscore, which represents the status of the tumoral immune system^39^ and the cytolytic activity as an in-silico metric of inflammation^40^. Analysis of miR-342-3p subgroups (low, intermediate and high) revealed a lower immunophenoscore values in the tumors with low and intermediate levels of miR-342-3p (Sup Fig. 3). These findings were confirmed by the markedly reduced values of the cytolytic activity in the low and intermediate subgroups (Sup Fig. 3). These results are in line with the above-described results. Overall, the data of this descriptive genomic analysis of human tumors suggested that the down-modulation of miR-342-3p is correlated with an increased aerobic glycolysis and metabolic phenotypes which promotes a immunosuppressive microenvironment in TNBC (Fig. 7d).

## Discussion

Accumulating evidence suggests that miRNAs play important roles in tumorigenesis and metabolic pathways. By a deep characterization of the miRNA transcriptional landscape of TN tumors, we identified a set of altered miRNAs, specifically miR-342-3p, which was down-regulated in TNBC compared with other phenotypes. MiR-342-3p is an intronic miRNA of the EVL host gene, which is also down-regulated in TN and basal tumors^13,41^, and present coordinated expression profiles^42,43^. In this study, we confirmed that the expression pattern of miR-342-3p is correlated with its host gene, and their transcription rate is regulated in part by the transcription factor activity of ER.

*In vitro* and *in vivo* studies have demonstrated that exogenous over-expression of miR-342-3p in colorectal, cervical, prostate, breast, hepatocellular and non-small cell lung cancer induced apoptosis and inhibited tumorigenicity, cell growth, invasion and migration, through the establishment of diverse cancer regulatory networks, suggesting a potential tumor suppressor activity^43–49^. In addition, experimental and network modelling data indicate that the enhanced expression of miR-342-3p results in chemoresistance reduction by repressing E2F^50^. Particularly in triple negative breast cancer, miR-342-3p has been reported as down-modulated, and its lower expression is associated with poor clinical outcomes^13,51,52^. In agreement with the literature, our *in vitro* assay on TN cell line models transiently over-expressing miR-342-3p resulted in decreased cell growth and viability rates, as well as reduced migration capabilities.

Our transcriptomic data identified specific pathways down-modulated by the exogenous expression of miR-342-3p that contributes to support key functions of TNBC cells such as gamma acid transports, fatty acid metabolism and cellular metabolism. An altered metabolism has been observed in different cancer types enhancing cancer cellular fitness to provide a selective biological advantage^21,22^. For instance, miR-342-3p may modulate metabolism through the direct post-transcriptional regulation of MCT1, an important lactate transporter^53^, which can facilitate the lactate flux between glycolytic and oxidative tumor cells^26,53–55^. The increased level of MCT1 is considered as hallmark of different malignancies and is associated with poor prognosis in the basal-like subtype^23,56^. A characterization of breast tumor metabolism has revealed heterogeneous intra-tumoral energetic pathways, where MCTs play a key role. For instance, lactate is introduced to cancer and non-cancer cells via MCT1, whereas MCT4 secreted lactate to the extracellular space^56,57^. The up-taken lactate is then converted into pyruvate to be incorporated into the Krebs cycle^58,59^. Under regular *in vitro* culture conditions, cancer cells show a metabolic symbiosis where a proportion of cells exhibit a glycolytic metabolism and other oxidative phosphorylation. We hypothesized that the exogenous expression of miR-342-3p in a glycolytic cell line model resulted in the down-modulation of MCT1, disrupting the metabolic fluxes and promoting the presence of more glycolytic cells. When MCT1 is inhibited, tumor cells consume more glucose than lactate as a fuel of source, breaking the metabolic symbiosis, and establishing an energetic competition which may trigger the reduced proliferative rates^53^.

In accordance with our results, it is reported that the inhibition of MCT1 by a pharmacological approach with AZD3965 resulted in a delay in tumor growth together with an increment of glycolysis in human tumor cell lines and cancer xenografts of small cell lung tumor. Importantly, a restriction in lactate uptake as a fuel source for metabolism was also observed^60^. This metabolic change forces the lactate consuming cells to use an alternative fuel, such as glucose, thus increasing the rate of glycolysis^60^. A similar switch from lactate-fueled respiration to glycolysis were observed in mouse models of colorectal adenocarcinoma and lung cancer cells after MCT1 inhibition by the administration of α-cyano-4-hydroxycinnamate^53^.

Moreover, in addition to glucose metabolism we also detected the over-representation of cholesterol metabolism in cells exogenously expressing miR-342-3p. Interestingly, among the possible mechanisms that promote the upregulation of cellular cholesterol synthesis, there is abundant availability of precursors such as acetyl-CoA generated via glycolysis^61,62^.

Little is known about the regulation of MCT1. Here we present how the post-transcriptional activity of miR-342-3p regulates MCT1 expression, as a direct target. Furthermore, it is also reported that metabolic conditions can impact the expression of MCT1 by glucose deprivation^37^. Consistently, our data demonstrate that miR-342-3p is also regulated by hypoxic and glucose deprivation conditions, favoring its down-modulation and the consequent over-expression of MCT1.

Finally, an enrichment pathway analysis in human TN tumors revealed a significant over-representation of glycolysis in tumors with low or intermediate expression of miR-342-3p. We propose that the down-modulation of miR-342-3p increases the expression of MCT1, leading to a higher cellular consume of lactate and promoting a more glycolytic phenotype, which is related with tumor aggressiveness. This idea is supported by the observations, made in different malignancies, that tumors with low levels of miR-342-3p and high levels of MCT1 are associated with poor clinical outcomes^18,63–66^. In accordance to the literature, tumors with the highest rates of glycolysis are more likely to have high MCT1 expression^67^, and we also show that tumors with low levels of miR-342-3p display an enrichment of glycolytic process. Recently, it has been reported that miR-342-3p modulated glycolysis by altering glucose uptake, lactate generation and extracellular acidification rate by suppressing IGF-1R-mediated PI3K/AKT/GLUT1 signaling pathway in hepatoma cells^68^.

Increasing evidence suggests that changes in tumor metabolism not only satisfies the energetic requirements of tumors cell, but also contributes to attenuate immune responses^69^, as we noticed in tumors with low levels of miR-342-3p and an increase in glycolytic signaling pathways. For instance, the metabolic interdependencies between tumor cells and immune cells result in metabolic competition, limiting proliferation and activities of tumor-specific immune cells. Moreover, metabolite abundance and accumulation of metabolic bioproducts such as lactate enhance a local immunosuppression^70–72^. Furthermore, lactate, the main bioproduct of glycolysis, inhibits the activity of antitumor immune cells effectors such as proliferation and activation of T and NK cells^73,74^, activation of monocytes and dendritic cell differentiation^75,76^ in *in vivo* and *in vitro* models of different human tumors. In accordance with these data, we detected a significant down-modulation of immune-related pathways as revealed by the reduced immunophenoscores and cytolytic activity in tumor over-expressing miR-342-3p with an increased glycolityc score.

Moreover, metabolic glucose fluxes negatively control cell adhesion mechanisms through specific pathways, such as integrin-dependent adhesion and migration, as reported by our enrichment pathway analysis on TNBC with reduced miR-342-3p levels. In addition, aerobic glycolysis may impact extracellular pH, which in turn controls integrin structure, cell adhesion and migration features^77,78^. Interestingly, we noticed that exogenous expression of miR-342-3p impairs the migration capability of MDAMB468 and BT549 cell lines, which are also two of the most glycolytic models within our set of cell models evaluated.

Altogether, our results suggest a connection between miR-342-3p and metabolic conditions in two distinctive scenarios: (1) The down-modulation of the tumor suppressor miR-342-3p in cancer programs triggers the establishment of a glycolytic metabolism and (2) exogenous expression of miR-342-3p in a glycolytic cell model disrupts lactate and glucose fluxes thus altering the metabolic established equilibrium. It is important to mention that tumors are open and adaptive systems sculpted by molecular changes over time, space and intrinsic relations between cancer and stromal cells. Molecular alterations of TNBC lead to tumor initiation and progression but also contribute to the establishment of metabolic networks, promoting for example a glycolytic phenotype or specific nutrient uptake pathways^79^. For instance, reported data showed that the over-expression of MYC supports glutamine metabolism in lung tumors, triggering biomass accumulation and cancer cell proliferation^80,81^. TP53 mutations increase glucose consumption and glycolytic flux, while inactivation of TP53 results in a higher dependence on serine uptake and metabolism^82^. Thus, we hypothesized that during early stages of carcinogenesis the oncogenic driven programs of tumors with high expression of miR-342-3p promotes a less glycolytic phenotype, in contrast the tumors with reduced expression of miR-342-3p presents an oncogenic context which favors glycolysis dependent pathways. Previous studies have suggested an important connection between glycolytic pathways, lactate production and tumor progression. Thereby, accumulation of lactic acid by highly glycolytic tumors has been described as a strategy for immune tolerance and evasion inducing an advantageous environment for tumor cells^73,75,83,84^. In this scenario, the oncogenic activity of the glycolytic metabolism might be explained by its immunosuppressive effects. On the other hand, in our *in vitro* approach we sought to take advantage of a glycolytic cell line, which recapitulates tumors with low levels of miR-342-3p and high rates of glycolysis, and where specific metabolic conditions are already well established. Consequently, when miR-342-3p is exogenously expressed, the immediate biological response disrupts the metabolic dynamic of the cells which increase glucose consumption to satisfy the new energetic demands, since they cannot use lactate because of the inhibition of MCT1 expression via the miRNA, and consequently a glycolytic process is promoted.

Loss of miR-342-3p expression and over-expression of MCT1 among tumor cells in TNBC contribute to the establishment of important pathways in tumor biology, thus these alterations open new possible therapeutics strategies for MCT1 inhibition that might have clinical implications. However, a deep clinical evaluation must be performed in animal models to determine how the altered expression of these molecules could be modulated to counteract oncogenic properties.

## Methods

### Cell line models

Human breast cancer cell lines were maintained in RPMI 1640 (Lonza) or DMEM (Gibco) medium supplemented with 10% fetal bovine serum (FBS, Gibco) at 37 °C in 5% CO. For hypoxic conditions the cells were grown on 1% oxygen atmosphere. All cell lines were authenticated by microstallite profiling and tested for mycoplasma.

### Human breast cancer tissues

Formalin fixed paraffin embedded (FFPE) tissues were collected from 164 patients from the pathological archival at Fundación para Enfermedades de la Mama FUCAM, Mexico and Istituto Nazionale dei Tumori, Milan (Italy). Tumor samples were obtained from each patient after informed consent for surgery, tumor resection, tissue collection and evaluation. Sample collection was approved by the committees for ethical review under institutional IRB approval protocol at National Institute for Genomic Medicine (INMEGEN), FUCAM and Istituto Nazionale dei Tumori. All procedures were carried out in accordance with national and international guidelines and regulations^85,86^. All the patients enrolled in the collection received adjuvant chemotherapy treatment. All tissue specimens were characterized by immunochemistry analysis. RNA from FFPE sections were extracted with dedicated kits to maintain small RNA fraction according to the suggested protocol (Recover All, Ambion).

### RNA extraction and quantitative RT-PCR analysis

Total RNA including small RNA fraction was isolated with Qiazol lysis reagent (Qiagen) according to the protocol suggested by the manufacturer. RNA retro-transcription was performed with the SuperScript III First-Strand Synthesis System (Invitrogen). Real-time PCR was carried out using TaqMan Gene Expression Assays (Applied Biosystems); for mRNA evaluation GAPH was used as an endogenous control whereas miRNA expression was evaluated with specific primers for cDNA synthesis and Taqman probes (Applied Biosystems) using RNU48 as endogenous control. All the experiments were performed on a Step One Plus system (Applied Biosystems) and data were processed by a DeltaCt method in R environment in the HTqPCR library.

### miRNA and mRNA expression profiling

miRNA expression profiles were processed with the oligo package^87^ implemented in Bioconductor of R environment. Microarrays for version 2 and 3 were background corrected by Robust Multichip Analysis (RMA) independently. Only common miRNA probes from version 2 and 3 microarrays were further processed. The complete cohort (N = 134) was then normalized all together by quantile method with the limma package. The signal intensities of the Human Transcriptome Array 2.0 were background corrected by RMA and normalized by quantile algorithm with the Transcriptome Analysis Console Software (Affymetrix). MiRNA and transcriptome normalized data were adjusted for batch effect by combat algorithm (Euclidean distance and non-parametric method) in the Genepattern platform (http://genepattern.broadinstitute.org/gp/pages/index.jsf)^88^ to avoid bias per batch effects. Differentially expressed profiles were computing with the limma package^89^ by a moderate t-test and adjusted p-value by FDR method.

### Data mining of TCGA and METABRIC data sets

Transcriptome and clinical data from The Cancer Genome Atlas (TCGA) were downloaded from the Xena browser (UCSC Xena http://xena.ucsc.edu) using the following cohorts: GDC TCGA Breast Cancer (HTSeq – Counts of gene expression RNAseq) and TCGA Breast Cancer cohort (miRNA mature strand expression RNAseq IlluminaHiseq). To evaluate TCGA data, low normalized counts (<10) data were filtered. Then, differentially expression profiles were computed using DESeq. 2^90^ on R environment adjusting p-values by false discovery rate (FDR) method. Normalized gene expression data from METABRIC were obtained from cbioportal^91^, gene differentially expression were determinate by limma package^89^ as previously described.

### MiRNA mimic and siRNA transient transfection

Transient transfection of chemically synthesized microRNA (miRNA) mimic hsa-miR-342-3p (Ambion, M12328) at final concentration of 30 nM was performed with Siport neoFX transfecion agent (Thermo Fisher) during 24 hr in Optimem (Gibco) medium according to the protocol suggested by the manufacturer. SiRNAs against ESR1 (Ambion, Id: s4824, s4823) were transfected at a final concentration of 50 nM with lipofectamine RNAimax transfection reagent during 24hrs in Optimem medium (Gibco) according to the manufacturer’s instructions. Cells were collected after 24, 48, 72, 96 and 144 hr upon transfection for further analysis.

### Functional analysis

Cell viability was assessed using the MTT Cell Proliferation Assay (ATCC). Briefly, cells were seeded in a 96-well plate at a density of 10000 cells per well. 48 hr after transfection the MTT reagent was added and incubated for 2 h at 37 °C. Absorbance was measured at 570 nm in Biorad spectrophotometric plate reader. The growth rate was assessed by using sulforhodamine B (SRB) at different time points (0, 24, 48,96, 120 and 144 h) 48 h post-transfection. The absorbance was read at 590 nm in a Biorad spectrophotometric plate reader. Migration assays were performed by a transwell system (Corning, 8um pore size). A density of 1 × 10^5^ cells post-transfection (48 h) were cultured in the upper chamber and incubated for 24 h. For the invasion assays, the transwell system was coated with 10% Matrigel (BD Biosciences) per chamber and incubated for 24 h. Migrated and invaded cells were fixed with 10% formaldehyde, stained with SRB and photographed for further quantification with imageJ. Apoptosis tests were performed with the Alexa Fluor® 488 Annexin V/Dead Cell Apoptosis Kit (Invitrogen) according to the suggested protocol, evaluating 1 × 10^6^ cells at 24 and 48 h post-transfection in a FACS Aria I cytometer (BD). Data were collected using FACSDiva analysis software (Beckman Coulter). Cell cycle phases were analyzed using CycleTEST PLUS DNA Reagent Kit (BD Biosciences) 48 h after transfection, and the cells were synchronized by serum starving. Fluorescence evaluation was performed in FACS Aria I cytometer (580 and 650 nm). Data were analyzed with modFit 3.2 software. Each experiment was performed in triplicate and independently repeated three times.

### Gene expression profiling of cell lines exogenously expressed miR-342-3p

Transcriptional landscape of MDAMB468 cell lines exogenously expressing miR-342-3p was evaluated with the Human Gene 1.0 ST microarrays (Affymetrix) following the manufacturer’s protocol and analyzed with the oligo and limma packages implemented in R. Briefly, background correction and normalization were performed with RMA and quantile method with the oligo library on Bioconductor. Duplicate probes were filtered by the maximum interquartile range (IQR), probes with maximum IQR were selected. Then, to detect miRNAs or mRNAs differentially expressed we applied a moderest t.test on the limma package. Genes with logFC of 0.7 and p.value < 0.05 were considered significantly differentially expressed.

### Pathway analysis

Comprehensive pathway enrichment analysis was performed with Enrichr^92^ data base using the Kegg, Reactome and biological process gene ontology annotation to map genes to each biological term. Adjusted p-values (Fisher test) denote the significance of the pathway enrichment (P value ≤ 0.05). Briefly, Enrichr computed a p-value adjustment by Z-score permutation background correction on Fischer exact test p-value, which assess the deviation from the expected rank. It also calculated a combine score by multiplying the unadjusted p-values with the z-scores. The combined score represents a robust ranking measurement of the enrichment of particular term.

### Implementation of ssGSEA

Individual scores for each tumor case were quantified by ssGSEA method implemented in Genepattern server (ssGSEA projection: https://genepattern.broadinstitute.org, parameters: min gene set size of 5, combine.add mode) using as gene set the annotation term of glycolysis (KEGG HSA00010) and anion transmembrane transport (GO:0098656). Briefly, ssGSEA is a rank-based method that computes an overexpression measure for a gene list of interests relative to all other genes in the genome^93^. We used normalized RNA-Seq (RSEM data) or microarray data log transformed to computed the ssGSEA scores. To combine the ssGSEA scores for the diverse cohorts analyzed, a z-score was computed to rank them in each tumoral cohort, then the transformed z-scored ssGSEA values were plotted all together in a unique boxplot. Statistical methods were applied to define a significance level between the distribution of the ssGSEA score level among each tumor subgroup.

### mRNA miRNA integrative analysis

We first computed the differentially expressed miRNAs and mRNAs of triple negative tumors vs non-triple negative tumors, as described above. Next, the post-transcriptional targets of each miRNA were predicted by a combinatory approach in mirWalk data base^94^ using the following algorithms: mirWalk, PITA, RNA22, miRanda, PICTAR2, miRDB and Targetscan. mRNAs predicted by at least 3 algorithms were maintained as true possible targets. Then, we performed a guilt-by-association analysis between the differentially expressed miRNAs and mRNAs previously predicted. Briefly, normalized profiles of the differentially expressed mRNAs and miRNA of the profiled tumors were used to build a correlation matrix (Spearman correlation and p value) in R. Each miRNA and mRNA were ranked according to their correlation coefficient and p value. A significant correlation between miRNAs and predictive mRNA was considered when Spearman correlation >30% and adjusted p value < 0.05. Finally, the correlated predicted targets with specific miRNAs were evaluated by an enrichment pathway analysis with the Enrichr tool^92^.

### Immunophenoscore determination

Immunophenoscore computed a score based on the gene expression values of immune-related genes to four classes: (1) effector cells (2) immunosuppressive cells, (3) MHC molecules and (4) selected immunomodulators^39^. The immunophenoscore values were determinate with the available R-script deposited on GitHub (https://github.com/mui-icbi/Immunophenogram) for our cohort and Metabric, while for TCGA the immunophenoscore was obtained from TCIA data base (https://tcia.at/home).

### Cytolytic activity

Cytolytic activity has been defined as reported by Rooney and collaborators^40^ as the geometric mean of GZMA and PRF1 normalized gene expression.

### Western Blot

Proteins were extracted with RIPA lysis buffer. The primary antibodies used were: MCT1 (Santa Cruz sc-365501, 1:200), HIF1a (BD, 6109591:200), Glut1 (Abcam, ab115730, 1:1000) and ER (Santa Cruz, sc-543, 1:200). Proteins were visualized by enhanced chemiluminescence detection system (GE Healthcare). Densitometry analysis was performed with ImageJ software.

### Dual-luciferase reporter assay

psiCHECK-2 (Promega) plasmid was used in the luciferase reporter assay. A 50 bp 3′UTR sequence of MCT1 mRNA containing the putative miR-342-3p-binding site and a mutated sequence were cloned in the plasmid. MDAMB468 cells were co-transfected with 450 ng of psciCHECK-3′UTR (WT or Mutated) and 30 nM of miR-342-3p precursor with Lipofectamine 2000. Firefly and Renilla luciferase expression were measured after 24 h with Dual-Luciferase Reporter Assay System (Promega).

### ***In vitro*** lactate and glucose measurements

Exogenous lactate consumption in MDAMB468 cells over-expressing miR-342-3p (2 × 105 cells) was assessed by adding 10 mM of exogenous lactate (L-lactate-3-13-c, Sigma) in DMEM free phenol glucose low media (1 mg/L, GIBCO) at different time points (0, 8, 24, 48, 72, 96, 120 h). Lactate concentrations were quantified by the colorimetric assay Lactate Assay I kit (Sigma), according to the manufacturer’s instructions. All the samples were de-proteinized with Amicon Centrifugal Filter Ultracel-10 kDa (Millipore). Intracellular and extracellular concentrations of transfected cell cultured in DMEM free phenol high glucose media (4 mg/L, GIBCO) were measured at different time points (24, 48, 72 h). Lactate was measured as previously described. Glucose concentration was assessed by Glucose Assay kit (Abcam). The glycolytic index was computed as the nmoles of lactate divided by the nmoles of glucose per time in each cellular compartment.

### Live cells optical redox ratio analysis

Stack images were obtained in a Leica SP8 confocal microscope from live cells transfected with miR-342-3p mimic or negative control using 40x oil-immersion objective (1.30 of numeric aperture). Stacks projections were acquired in 2 mm depth increments with a median time for image acquisition of 20 seconds with 10 planes per point. The average intensity laser for NADH was 452 nm (range 410–495, channel 1), while for FAD 768 nm (range727–733, channel 3). Fluorescence analysis was performed in ImageJ as following: to minimize shot noise across stack frames, we first applied Kalman stack Filter (acquisition noise variance estimation: 0.05 and bias to be placed on the prediction = 0.60). Then, to subtract local background intensity we applied the rolling ball method according to the average cell ratio. Then the integrative total intensity of FAD and NADH was computed by a z-projection with sum slices type. The redox ratio image was thresholding to remove background with subtract background function and finally the fluorescence was measured. All the images were analyzed as 8 bit, only for figure purposes we used RGB color type. The optical redox ratio was computed from the NADH and FAD fluorescence data as following: Redox ratio = NADH/FAD.

### Statistical analysis

Statistical analysis to define significant differences between the experimental conditions was performed by Wilcoxon test or Kruskal-Wallis, to compare two groups or multiple groups respectively (non-parametric), using R software or Graph Pad Prism 7 software (GraphPad software Inc., San Diego, CA). A confidence value of 95% (p-value ≤ 5%) was considered significant. P-value: * < = 0.05, ** < = 0.01, *** < = 0.001, **** < = 0.0001. For microarray data, a moderated t-test was implemented and false discovery rate (FDR) multiple adjusted test was computed to define the adjusted p-values on limma library of Bioconductor environment.

### Available data

Data reported in this paper have been deposited in GEO: miRNA profiles from tumors GSE86281, GSE86278, GSE86277; mRNA profiles from tumors: GSE86946, GSE86945 and mRNA profiles from cells exogenously expressed miR-342-3p: GSE86948. All the data conforms the Superseries GSE86948.

## Electronic supplementary material

Supplementary Figures

Supplementary table 1

Supplementary table 2

Supplementary table 3

Supplementary table 4
