# Supplementary material for: Loss of function of miR-342-3p results in MCT1 over-expression and contributes to oncogenic metabolic reprogramming in triple negative breast cancer
Source: Sci Rep. 2018 Aug 16;8:12252. doi: 10.1038/s41598-018-29708-9 (PMC6095912; doi:10.1038/s41598-018-29708-9)
Supplement: Supplementary file 1 — Supplementary Figures [file 41598_2018_29708_MOESM1_ESM.pdf]

Loss of function of miR-342-3p results in MCT1 over-expression and contributes to oncogenic metabolic reprogramming in triple negative breast cancer

Sandra L. Romero-Cordoba[1,3], Sergio Rodriguez-Cuevas[2], Veronica Bautista-Pina[2], Antonio Maffuz-Aziz[2], Elvira D'Ippolito[3], Giulia Cosentino[3], Sara Baroni[3], Marilena V. Iorio[3]*, Alfredo Hidalgo-Miranda[1]*.

Supplementary figures

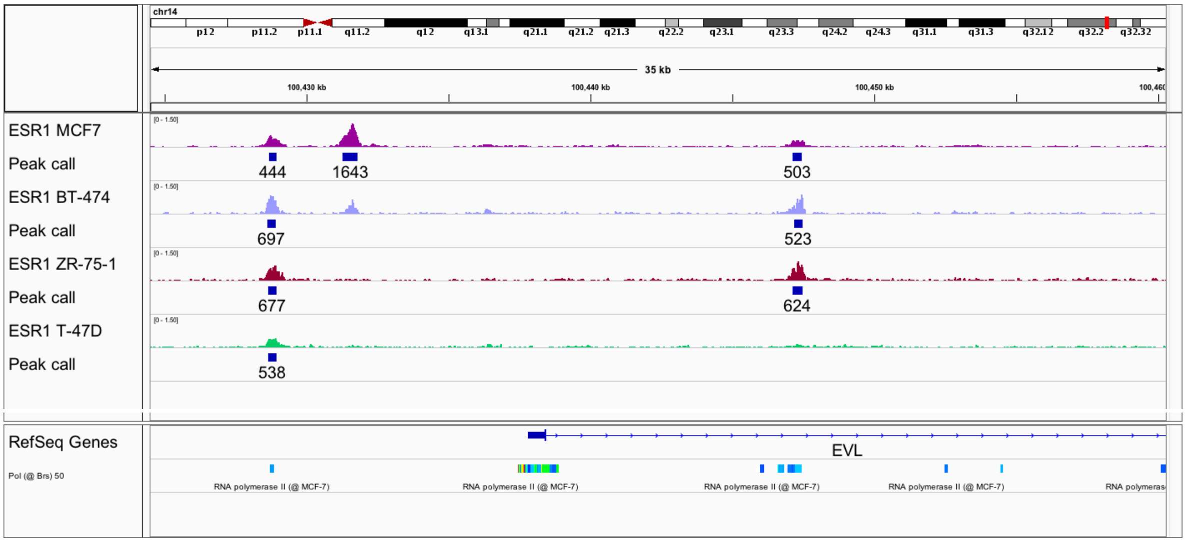

Supplementary Figure 1: ER (ESR1) chip-seq data from ER+ cell lines on EVL upstream region. ESR1 peaks identified by manual analysis on IGV. Upper track corresponds to sequence reads identified in 1kb upstream EVL region, lower track shows the average coverage for each peak. The second lower panel shows the genomic position of EVL and Pol II occupancy. All chip-seq data were retrieved from chip-atlas data base.

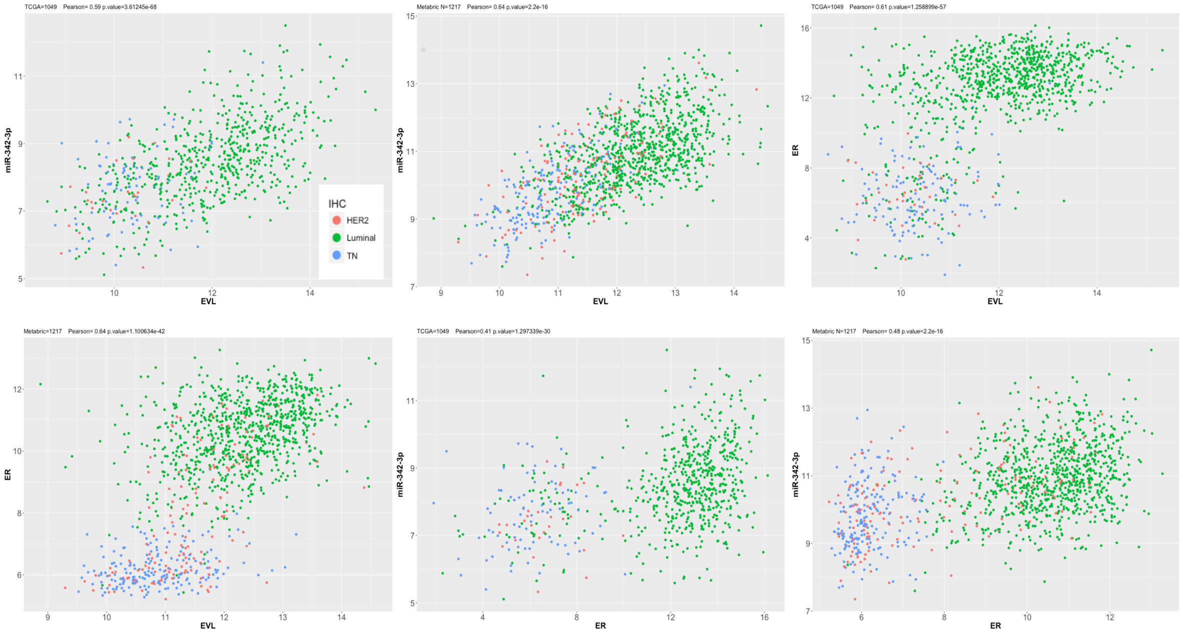

Supplementary Figure 2: miR-342-3p expression negatively correlated with the transcriptional status of ER and its host gene EVL. Scatterplot of the expression of miR-342-3p and its host gene across breast cancer samples in (a) TCGA and in (b) METABRIC data bases. Scatter plots showing a significant anti-correlation between the expression level of EVL and ER in tumor samples of (c) TCGA and in (d) METABRIC, as well as miR-342-3p and ER in (e) TCGA and in (f) METABRIC. Pearson correlation coefficient and significant value are indicated in the plot.

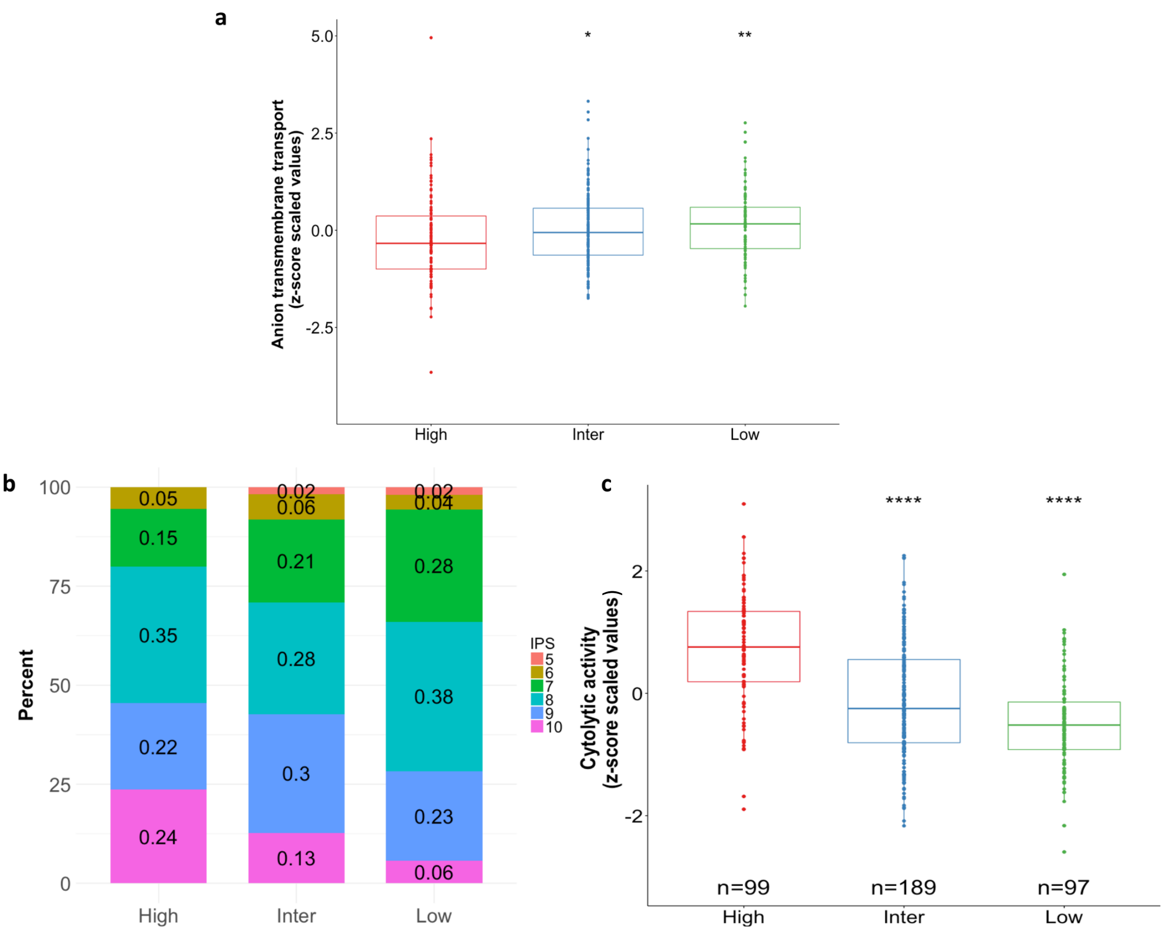

Supplementary Figure 3: Lower miR-342-3p expression is characterized by enrichment of anion transmembrane transport and immunosuppressive phenotype. a) Z-score values of single sample GSEA analysis (ssGSEA) scores based on anion transmembrane transport term of TN tumors from our cohort, METABRIC and TCGA database stratified by miR-342-3p expression: low (≤1st quantile), intermediate (>1st quantile but < 3rd quantile) and high (≥ 3rd quantile). b) Immunophenoscores (IPS) of our cohort and TCGA and c) cytolytic activity of our cohort, Metabric and TCGA of each tumor group divided by miR-342-3p expression.

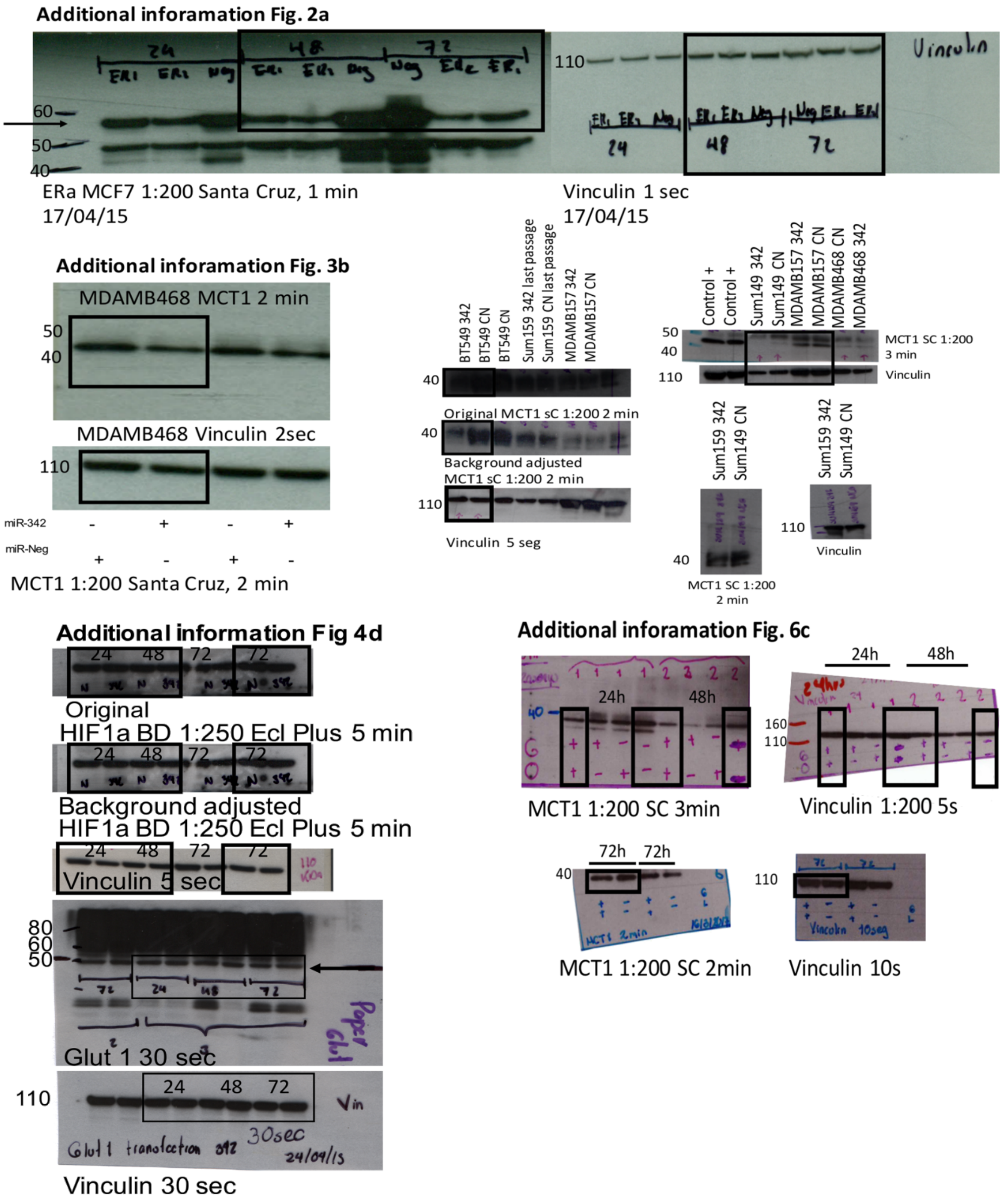

Supplementary Figure 4: Additional information of western blot plots shown in the paper

Supplementary tables

Table S1: miRNA expression profile of TN tumors vs other phenotypes (ER+, PR+ and/or Her2+)

Table S2: Integrative analysis of miRNA and mRNA expression in TN tumors and enrichment pathway analysis of significantly correlated miRNA-mRNAs.

Table S3: Gene expression profile of MDAMB468 cell line exogenously expressing miR-342-3p and enrichment pathways analysis of differentially expressed genes.

Table S4: Gene expression profile of human TN tumors expressing diverse miR-342-3p levels (Low vs High) and the enrichment pathways analysis of differentially expressed genes.
